# Pregnancy Outcome in Cartilage-Hair Hypoplasia, a Rare Form of Dwarfism

**DOI:** 10.1155/2017/4737818

**Published:** 2017-01-31

**Authors:** Harshithaa Thavarajah, Anne Berndl

**Affiliations:** ^1^University of Queensland, Brisbane, QLD, Australia; ^2^Sunnybrook Health Sciences Centre, Toronto, ON, Canada; ^3^Department of Obstetrics and Gynecology, University of Toronto, Toronto, ON, Canada

## Abstract

*Background*. This case report discusses the pregnancy outcome of a patient with cartilage-hair hypoplasia, a rare form of dwarfism, and multiple previous orthopedic surgeries. Literature on pregnancy outcomes in patients with cartilage-hair hypoplasia is limited.* Case*. A 32-year-old patient with cartilage-hair hypoplasia presented at 12 weeks' gestation to the high-risk obstetrics clinic for care. Preterm labor resulted in cesarean delivery at 34 weeks' gestation with general anesthetic. Breastfeeding was stopped at 6 weeks due to neonatal complications.* Conclusion*. Pregnancy and delivery were uncomplicated. A multidisciplinary approach allowed for effective management during pregnancy and postnatal care. This is the first known documented case of prenatal care, delivery, and breastfeeding in a woman with this rare disorder.

## 1. Introduction

### 1.1. Overview of Short Stature, Dwarfism, and Cartilage-Hair Hypoplasia

Dwarfism has been defined as individuals with a height less than 148 cm at adulthood [1]. There are over 100 [2] forms of dwarfism that have been documented; however there is limited information surrounding pregnancy, birth, and breastfeeding in this population. The majority of documented experiences focus on anesthetic management [1, 3–6]. Cartilage-hair hypoplasia (CHH) is a rare form of short-limbed dwarfism with an incidence of 1/23,000 births [7]. It is the result of a genetic autosomal recessive pattern of inheritance, from a mutation to the noncoding RNA gene, RMRP [8]. The RMRP mutation disrupts ribosomal processing and progression through the cell cycle in rapidly dividing cells, such as chondrocytes [8], which results in defective cell proliferation [7]. Cartilage deficiency at the metaphyseal ends of bones causes shortening of limbs [8]. Similar disruption to lymphocytes can result in defective proliferation of T lymphocytes, causing features of severe T cell immunodeficiency [8]. Other associated clinical features include sparse hair growth, bone marrow failure, increased risk of Hirschsprung disease, and malignancies [8]. It is a disease of highly variable penetrance with phenotypic differences evident within families.

There have been few case reports in patients with cartilage-hair hypoplasia and other forms of dwarfism, discussing their clinical manifestations, treatment, and genetic implications; however, pregnancy outcomes in these patients have not been published. A survey of obstetrical and gynecological outcomes in women with chondrodystrophies in 1986 included 3 women with CHH [9]; however details of their pregnancy are unknown. There have been case reports in the literature on the pregnancy outcomes in patients with diastrophic dwarfism [5], achondroplasia [1, 3], pituitary dwarfism [10], spondylometaphyseal dysplasia [4], and osteogenesis imperfecta [11], with a substantial risk of cesarean delivery. Of these, many focused on the anesthetic planning for their cesarean deliveries. Breastfeeding rates or concerns were not mentioned. These patients tended to suffer from respiratory distress towards the end of their pregnancy. A main concern in patients with varying forms of dwarfism is the use of anesthetics, both general and regional dosing can be problematic.

Pregnancy loss and fetal complications do appear to be increased in maternal dwarfs [12, 13]; however this is not seen in all studies [14]. From the obstetric view, women of short stature are also at increased risk of preterm birth [14] and have been found in multiple studies to have a higher risk of cesarean delivery, often associated with cephalopelvic disproportion [12, 14]. However, in patients with skeletal dysplasias with less disproportion, vaginal deliveries have been noted [14].

It should be noted that what is defined as short stature may differ between ethnic populations. In populations with lower average maternal height, there is proportionately less impact of height on obstetrics [13].

## 2. Case Report

A 32-year-old gravida 3, para 0, was referred at 13 weeks 5 days' gestation to the High-Risk Clinic in June 2015 due to her maternal skeletal dysplasia, cartilage-hair hypoplasia. This diagnosis was established based on clinical findings and confirmed by genetic studies. She is 4 ft 0 in (122 cm) tall and has had eleven surgeries of the spine with Harrington rods placed and 10 inches (25.4 cm) of lower limb lengthening. She had no ongoing musculoskeletal concerns. She has also had bilateral breast implants. Her obstetrical history included a first-trimester miscarriage of a spontaneous pregnancy and an ectopic pregnancy following IVF, treated with methotrexate. This pregnancy occurred spontaneously. She has no history of gastrointestinal manifestations, immunodeficiency, or autoimmune disorders, which can be associated with cartilage-hair hypoplasia; however, she reports a history of sciatica. Informed consent was obtained from the patient in writing this case report.

Genetic counseling had been provided to the patient and her unaffected partner prior to conception and he was not found to be a carrier. As a result of her extremely short stature and associated skeletal abnormalities, elective cesarean at term was recommended. Baseline pulmonary function tests were done and a referral to consultation with anesthesia was made during the second trimester. Due to her previous surgeries along the spine, the decision was made for general anesthesia. Cervical length was monitored from 16 weeks' gestation due to increased risk of preterm birth and measured to be 3.5–3.8 cm. Pulmonary function tests were repeated at 31 weeks' gestation and remained normal. Her 20-week anatomy scan showed a persistent right umbilical vein and midline gallbladder, and fetal echo showed a single papillary muscle of the fetal mitral valve. She opted for noninvasive prenatal testing, which was normal.

Concerns were raised regarding increased intraabdominal pressure and potential difficulties acquiring adequate nutrition, respiratory compromise, preterm birth and intrauterine growth restriction, difficulty in ambulation and instability, and anesthetic management. She experienced some back discomfort and occasional pain in the left hip, which was managed with Tylenol, physiotherapy, and massage. Occupational therapy was also suggested; however the patient had improvement with swimming and therefore did not have this consult. She was also advised on hydration, stretch exercises, and increased dairy intake to help with nocturnal leg cramps. Referral was made for dietician review every 2 weeks to monitor her progress with weight gain and associated symptoms relating to constipation. The lack of evidence regarding ideal body weight and gestational weight gain for people of short stature was discussed. She gained a total of 30 lbs during her pregnancy, which is within the normal weight gain recommended by the Society of Obstetricians and Gynecologists of Canada for women of normal BMI [15]; however, she experienced satiety early on and reported eating slower. Increased fruit and vegetable consumption was recommended, with smaller portions overall. She was on docusate and increased fluid intake was encouraged to improve constipation. Fiber content was not increased to avoid further limiting her hunger and weight gain.

Because of her desire to breastfeed and the possible associated difficulties due to her short upper limbs, she had a prenatal lactation consultation. It was discovered that her short upper limbs may limit her movement, and she was therefore provided with teaching surrounding laid back positioning, which involves lying back at a 45-degree angle and allowing the baby to use their reflexes to “bob” on and off until finding the breast. The concern regarding reaching the breasts for pumping was addressed by recommending a sports bra with holes cut in it, and using this to keep the breast pump flanges in place.

She presented to hospital at 34 weeks 2 days' gestation with fetus in breech position and active labor following spontaneous rupture of membranes. An uncomplicated cesarean was performed with a low segment transverse abdominal incision and general anesthesia followed by an uneventful postoperative recovery. She gave birth to a male infant weighing 2.4 kg. She was discharged 2 days later with support from the hospital breastfeeding clinic. She breastfed for approximately 6 weeks but stopped due to neonatal cow's milk allergy.

## 3. Discussion

### 3.1. Maternal Complications

Obstetric and gynecologic complications have been sparsely studied in the population of women with short stature and different forms of dwarfism. Preterm deliveries frequently occur due to maternal respiratory distress [9]. In this case, respiratory disease was not a primary concern; however, pulmonary baseline testing was conducted and shown to be normal. Due to the normal fetus head size in a smaller maternal pelvis [1], this can cause cephalopelvic disproportion and result in dystocia [12]. As a result, elective cesarean deliveries are sometimes recommended, as it was in this case. Short stature has been shown to be an independent risk factor for cesarean delivery [12].

During pregnancy and labor, there is the added concern of appropriate pain management, particularly in these patients who have had multiple previous surgeries due to orthopedic problems. Continuous spinal epidural anesthesia has been successfully used during labor in women with scoliosis in previous spinal surgery [5] and people with achondroplastic dwarfism, even in urgent cesarean deliveries. Regional anesthesia may be difficult due to poor landmarks, spinal deformity, and narrowing of the epidural spaces, which increases the risk for dural puncture [5]. With regional anesthesia, neurological complications have not been reported in this cohort of patients [1]; however, since this patient reported a history of sciatica in addition to her history of spinal surgeries, general anesthesia was chosen. General anesthesia has been recommended in some cases [9] but may require postoperative ventilator support [5]. There is insufficient data regarding pain management during the duration of pregnancy, particularly in these patients who may have increased pain secondary to skeletal problems and multiple previous surgeries. Nerve-root compression has also been reported as a complication in the short stature literature [16]; however this patient did not suffer from this difficulty.

Referral was made to a dietician during pregnancy to assess the appropriate weight gain, BMI, and nutrition, given her short stature. There is a lack of literature on this topic; however it is postulated that her increasing intraabdominal pressure may have contributed to her constipated bowel movements and pelvic discomfort.

### 3.2. Fetal Complications

Genetic testing was performed and appropriate genetic counseling is important in patients with cartilage-hair hypoplasia, which is an autosomal recessive condition [8].

Many studies have shown the association between maternal short stature with increased risk of premature labor [13] and preterm births [16]. The risk of preterm births appears to be secondary to both spontaneous labor [16] and respiratory distress [5]. Fetal growth in women with dwarfism can encroach on the diaphragm in later stages of pregnancy [1], causing dyspnea and necessitating an earlier date of delivery, often with the use of corticosteroids to optimize fetal lung development.

In women with cartilage-hair hypoplasia, the anteroposterior diameter of the pelvic inlet can be shorter, pushing the uterus up so that it becomes an intra-abdominal organ earlier in pregnancy [9]. As a result of this limited ability for uterine expansion in patients with relatively shortened trunks [9], there is potential for intrauterine growth restriction. The birthweight of infants born to women with short stature overall has been found to be about average; however infants of women with cartilage-hair hypoplasia appear to have slightly lower birthweights [9].

### 3.3. Postnatal Complications

Our primary postnatal concern was the ability to breastfeed given her shorter upper limbs; however, she was able to express immediately after delivery. Data is lacking on breastfeeding in women with dwarfism. Early referral to the breastfeeding clinic was made as a result of the patient's goal to breastfeed for at least 2 years. Here, the benefits of early skin-to-skin and hand expressing were discussed. Although concerns had been raised about finding an appropriate position for breastfeeding according to the limits of her arms' length, this patient breastfed successfully for 6 weeks, stopping not because of her ability to breastfeed but on recommendation from her pediatrician due to the baby being diagnosed with cow's milk protein allergy, a condition whereby cow's milk protein from the mother's diet passes into the breast milk and causes an allergic and gastrointestinal reaction in the neonate [17].

## 4. Conclusion

Overall, this patient with cartilage-hair hypoplasia had reasonable outcomes. She avoided the major complication of pulmonary dysfunction and had an emergency cesarean delivery in active preterm labor after spontaneous ruptured membranes at 34 weeks. Primary concerns during pregnancy related to respiratory function, preterm birth risk, and appropriate weight gain. She successfully breastfed her infant with the support of the breastfeeding clinic for the first 6 weeks of life, which is especially important in the context of preterm birth.

## Additional Points

*Teaching Points*. (1) The pregnancy and delivery of this patient with cartilage-hair hypoplasia were effectively managed using a multidisciplinary approach. (2) Since patients with dwarfism are at increased risk for preterm birth, which may be secondary to maternal respiratory distress, pulmonary function was monitored during pregnancy. This patient did not have any respiratory complications and spontaneously labored resulting in cesarean delivery at 34 weeks' gestation, possibly secondary to short stature. Monitoring for preterm births and respiratory function is important in this population. (3) Despite having breast surgery and short upper limbs, she was able to breastfeed with the help of a team of lactation consultants. Data on breastfeeding in women with dwarfism is lacking; however, it can be done with the appropriate support.
